# The efficacy and safety of patient-specific instrumentation versus conventional instrumentation for unicompartmental knee arthroplasty: Evidence from a meta-analysis

**DOI:** 10.1097/MD.0000000000036484

**Published:** 2024-01-19

**Authors:** Huihui Wang, Liang Zhang, Xueren Teng

**Affiliations:** a Qingdao Eighth People’s Hospital, Qingdao, China.

**Keywords:** knee osteoarthritis, patient-specific instrumentation, unicompartmental knee arthroplasty (UKA)

## Abstract

**Background:**

The aim of this study was to compare the efficacy and safety of patient-specific instrumentation (PSI) and conventional instrumentation (CI) for unicompartmental knee arthroplasty. Our hypothesis was that the PSI would be superior to CI in improving implant positioning and clinical function.

**Methods:**

We searched electronic databases (PubMed, Web of Science, Embase, and Cochrane) to identify relevant studies published before July 1, 2023 that met our inclusion criteria. The identified reports at least included one of the following outcome variables: coronal component alignment, sagittal component alignment, number of outliers, hip-knee-ankle angle, postoperative complications, operative time and knee joint functional evaluation. For dichotomous variables, we calculated the risk ratio and its 95% confidence interval (CI). For continuous variables, we calculated the mean difference (MD) and its 95% CI. Heterogeneity of the included studies was assessed using the standard chi-square test. Meta-analyses were performed using RevMan 5.4. software. The meta-analysis was registered with PROSPERO (No. CRD42023454160).

**Results:**

A total of 9 articles were included in the analysis, consisting of 4 randomized controlled trials and 5 cohort studies. The study population comprised 494 patients, with 262 in the PSI group and 232 in the CI group. Our findings demonstrate that the PSI group exhibits superior tibial component coronal alignment compared to the CI group (MD = −0.66, 95% CI: −1.21 to −0.12, *P* = .02). Conversely, the CI group demonstrates better femoral component coronal alignment than the PSI group (MD = 0.89, 95% CI: 0.17–1.60, *P *= .01). No significant between 2 groups differences were observed in tibial component sagittal alignment, femoral component sagittal alignment, tibial coronal axis outliers, tibial sagittal axis outliers, femoral coronal axis outliers, femoral sagittal axis outliers, postoperative complications, operative time, hip-knee-ankle angle, and postoperative knee joint function score.

**Conclusions:**

Our study findings suggest that the PSI confer an advantage in achieving superior tibial component coronal alignment, whereas the CI associated with better femoral component coronal alignment. However, no significant differences were observed between the groups in terms of other parameters. Future studies with larger sample sizes are needed to validate these findings.

## 1. Introduction

Knee osteoarthritis is one of the most common joint diseases worldwide, which is characterized by knee pain and structural changes, leading to resulting in disability, diminished quality of life, and financial burden.^[1]^ Joint replacement surgery is the primary surgical treatment for knee osteoarthritis at present. Compared to traditional total knee arthroplasty, unicompartmental knee arthroplasty (UKA) offers advantages such as faster postoperative recovery, improved postoperative range of motion, preservation of bone stock, lower mortality rate, and fewer complications.^[2,3]^ Despite various advantages, higher revision surgery rates continues to challenge patients who treated with UKA.^[4]^ Therefore, decrease the risk of revision surgery for patients who underwent UKA has become an important issue to be solved urgently.^[5]^

Previous studies have found that poor positioning of the components is an important factor associated with the higher revision rate of UKA.^[6,7]^ In recent years, many researchers are also actively exploring how to solve some of the abovementioned problems. The use of patient-specific instrumentation (PSI) for UKA is an innovative surgical technique that uses imaging techniques and 3D printing to reconstruct a 3-dimensional model of the patient bone tissue to meet the specific needs of the patient.^[8,9]^ The 3D model is utilized to determine the extent of femoral and tibial bone resection, as well as the size and positioning of the prosthesis, facilitating preoperative simulation and surgical planning.^[10]^ Theoretically, this approach has the potential to reduce the incidence of malalignment of the lower limb and improve postoperative knee joint function.^[9,11]^ However, the advantages of routinely implementing PSI in UKA continue to be controversial.^[12–15]^ The selection of effective approach is a conundrum that has always clinicians. In light of this, a systematic review is needed to comprehensively understand the efficacy and safety of PSI versus CI in UKA to guide clinical decisions.

**Figure 1.**
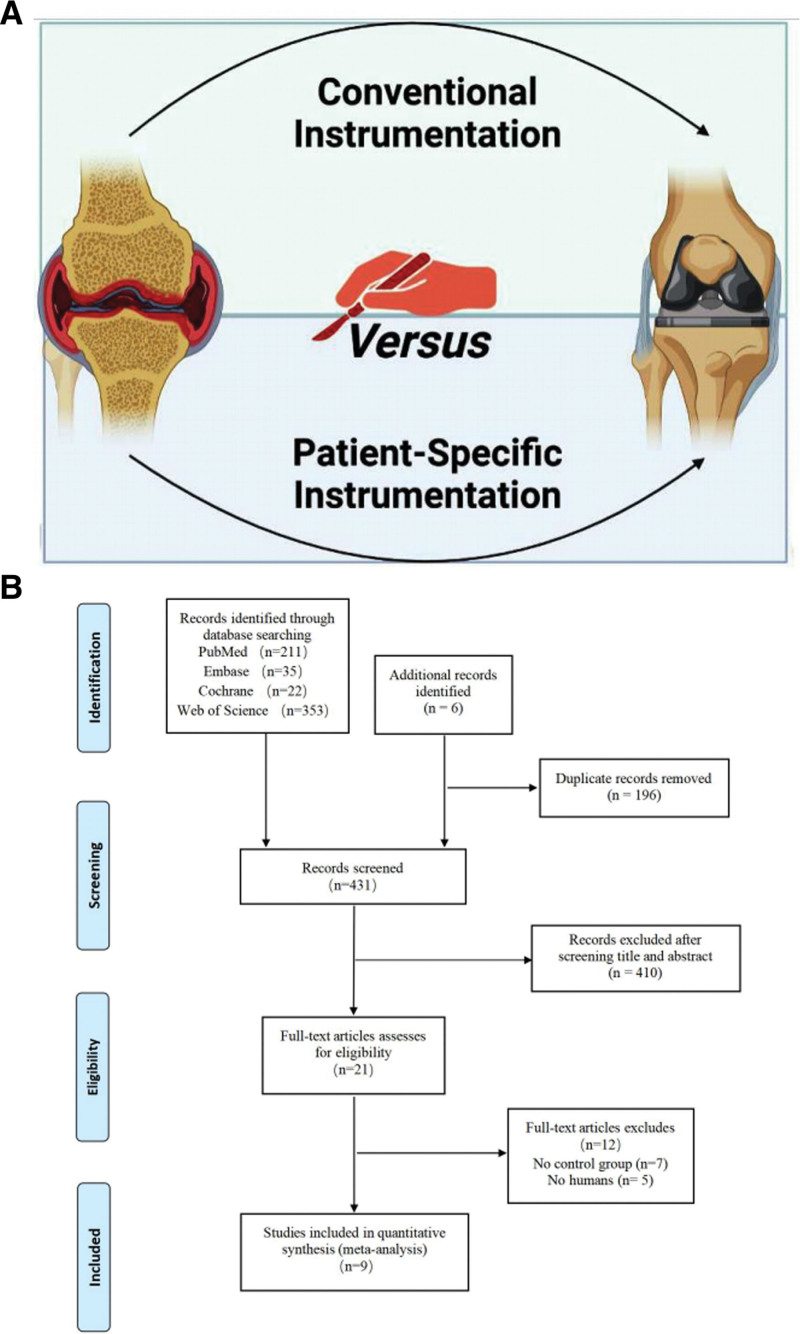
(A) Study design of the present study. (B) Flow diagram shows the process of selecting studies to be included in the review.

The utilization of PSI in UKA is a pilot attempt, but the clinical application value lacks the support of higher-level data. As far as we know, there has been no meta-analysis of previous studies reporting the advantages and shortcomings of PSI compared to the CI for UKA, which could to help guide clinical decisions. Therefore, we performed a meta-analysis of the available studies to compare the efficacy and safety of PSI versus CI for UKA. Figure 1A illustrates the purpose of the design of this study. Our hypothesis was that the PSI would be superior to CI in improving implant positioning and clinical function.

## 2. Methods

This meta-analysis was conducted according to the Preferred Reporting Item of Systematic Reviews and Meta-Analysis statement for this study.^[16]^

### 2.1. Search strategy

According to the Cochrane Collaboration guidelines, a systematic and comprehensive search was conducted in 4 databases: PubMed, Embase, Cochrane Library, and Web of Science. Additionally, a secondary manual search was performed on selected reference lists to identify any experiments that may have been missed during the database search. All inquiries were conducted using specific keywords, including UKA, 3D printing, patient-specific instruments, patient matching, custom instruments, and custom cutting blocks. Two independent reviewers screened titles, abstracts, and full texts from both electronic and manual searches. Any discrepancies during the process were resolved through discussion at meetings. Our detailed search strategy can be found in the attachment named “Electronic Search Strategy”, Supplemental Digital Content, http://links.lww.com/MD/L291.

### 2.2. Inclusion and exclusion criteria

Criteria for inclusion included: Study population: The experimental group received PSI while the control group used CI, with no specific restrictions on the type of prosthesis. Study design: Randomized controlled trials and non-randomized studies, including non-randomized controlled trials and prospective or retrospective cohort studies. Outcome indicator: the primary outcomes were the coronal component alignment, sagittal component alignment, number of outliers and hip-knee-ankle angle; the secondary outcomes were postoperative complications, operative time and knee joint functional evaluation. Exclusion criteria are as follows: Biomechanical experiments, animal studies. Reviews, letters, or conference abstracts. Duplicate publications and articles that cannot be accessed in full text. Articles not published in English. Studies with low quality.

### 2.3. Quality assessment

Two authors conducted a quality assessment of the included RCTs and prospective studies, and any discrepancies in quality assessment were resolved by a senior author. The methodological quality of the included RCTs was assessed using the Risk of Bias Assessment tool recommended by the Cochrane Collaboration.^[17]^ It includes the following 6 aspects: random sequence generation; allocation concealment; blinding of participants and personnel; completeness of outcome data; selective reporting of results; and other sources of bias. The assessment of these 6 items is categorized into 3 levels: low risk of bias, high risk of bias, and unclear risk of bias.

Non-randomized studies were carefully examined using the validated Methodological Index for non-randomized studies (MINORS) criteria for quality assessment.^[18]^ According to the MINORS criteria, comparative studies can receive a maximum score of 24. The quality categories for comparative studies are as follows: scores 0 to 6 indicate very low quality, scores 7 to 10 indicate low quality, scores 11 to 15 indicate moderate quality, scores 16 to 20 indicate high quality, and scores ≥ 20 indicate very high quality.

### 2.4. Data extraction

Two authors independently extracted the included literature data. When differences arise, the agreement is reached through discussion between authors. When the reported data is insufficient, we try to contact the author to obtain the raw data. The following data were extracted: first author, publication year, patient demographics, coronal and sagittal component alignment, hip-knee-ankle angle, postoperative complications, number of outliers, operative time and assessment of knee joint function.

### 2.5. Statistical analysis

Statistical analysis was performed using RevMan 5.4 software (Cochrane Collaboration, Copenhagen, Denmark). For dichotomous variables, risk ratio (RR) and its 95% confidence interval (CI) were calculated. For continuous variables, mean difference (MD) and its 95% CI were calculated. Heterogeneity of the included studies was assessed using the standard chi-square test. If the *P* value of the chi-square test was <.1 and *I*² was >50%, the data were considered to have high heterogeneity, and a random-effects model was used for analysis. In addition, a fixed-effects model was used for analysis. A *P* value of <.05 was considered statistically significant for differences.

## 3. Results

### 3.1. Literature search

A total of 627 articles were identified through searches across 4 databases. After removing 196 duplicate articles, screening of titles, abstracts, and full texts was conducted according to the inclusion criteria. Ultimately, 9 studies^[19–27]^ met the quality assessment and were included for data extraction. The process of study selection and inclusion is shown in Figure 1B. All included studies were published between 2015 and 2022, involving a total of 494 patients (262 in the PSI group and 232 in the CI group), with an average age range of 56 to 71 years. The basic characteristics of the included studies are presented in Table 1.

**Table 1:** Characteristics of the included studies.

Author	Study design	Pre-imaging	PSI system	Gender (F/M)		BMI	
				PSI	CI	PSI	CI
Demange et al (2015)	Prospective cohort study	MRI/CT	Zimmer, Warsaw, IN, USA	21/11	10/9	28.7 (20.7–41.7)	32.7 (26.5–46.5)
Kerens et al (2015)	Prospective cohort study	MRI	Biomet, Bridgend, UK	17/13	17/13	29 (23–40)	29 (20–37)
Ollivier et al (2016)	RCT	MRI	Zimmer, Warsaw, IN, USA	17/13	17/13	27 (20–31)	28 (22–33)
Alvand et al (2018)	RCT	MRI	Zimmer Biomet Inc, Warsaw, IN, USA	13/10	9/13	29.8 (23.8–40.3)	31.8 (22.2–39.5)
Jones et al (2019)	Prospective cohort study	CT	Embody, London, UK	10/20	7/7	NR	NR
Sanz-Ruiz et al (2019)	Prospective cohort study	MRI	Zimmer Biomet Inc, Warsaw, IN, USA	17/8	18/7	28.8 (21–34)	29.5 (23–35)
Gu et al (2020)	RCT	MRI/CT	Zimmer Ltd., USA	8/3	6/5	NR	NR
Kalache et al (2020)	Prospective cohort study	CT	NR	6/19	15/7	27.3 ± 2.9	26.4 ± 3.6
Leenders et al (2022)	RCT	MRI	Zimmer Biomet Inc, Warsaw, IN, USA	35/21	29/30	29 ± 4.3	29 ± 4.5

CI = conventional instrumentation, CT = Computed Tomography, F = female, M = male, MRI *=* magnetic resonance imaging, NR *=* not reported, PSI *=* patient-specific instrumentation, RCT *=* randomized controlled trial.

### 3.2. Quality and risk of bias assessment

**Figure 2.**
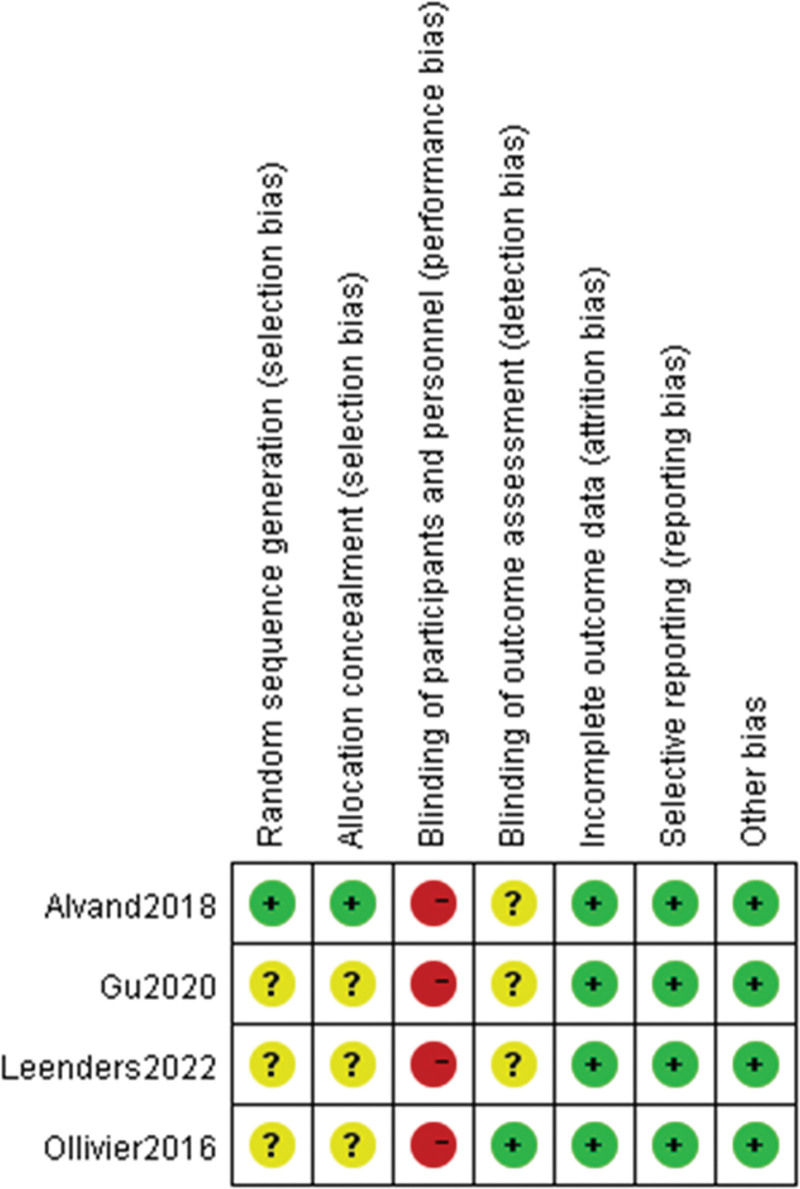
Risk of bias for each randomized study. Color represents the quality of each domain (red = high risk, yellow = uncertain, green = low risk).

Most RCTs described relatively objective outcomes. There were 3 RCTs where the random sequence generation and allocation concealment were unclear. Additionally, due to the nature of comparing 2 different surgical approaches, it was not feasible for the surgical teams to be blinded, leading to a high risk of bias from participants and personnel across all studies. Detailed information on the risk of bias for RCTs is shown in Figure 2. All non-RCTs were evaluated according to the MINORS criteria for comparative studies, with a maximum subjective score of 24. Table 2 provides a graphical representation illustrating the scores.

**Table 2:** The methodological index for non-randomized studies (MINORS) criteria.

Author	Study design	MINORS score out of 24
Kerens et al (2015)	Prospective cohort study	19
Demange et al (2015)	Prospective cohort study	18
Sanz-Ruiz et al (2019)	Prospective cohort study	20
Jones et al (2019)	Prospective cohort study	17
Kalache et al (2020)	Prospective cohort study	20

### 3.3. Postoperative tibial coronal alignment and number of outliers

**Figure 3.**
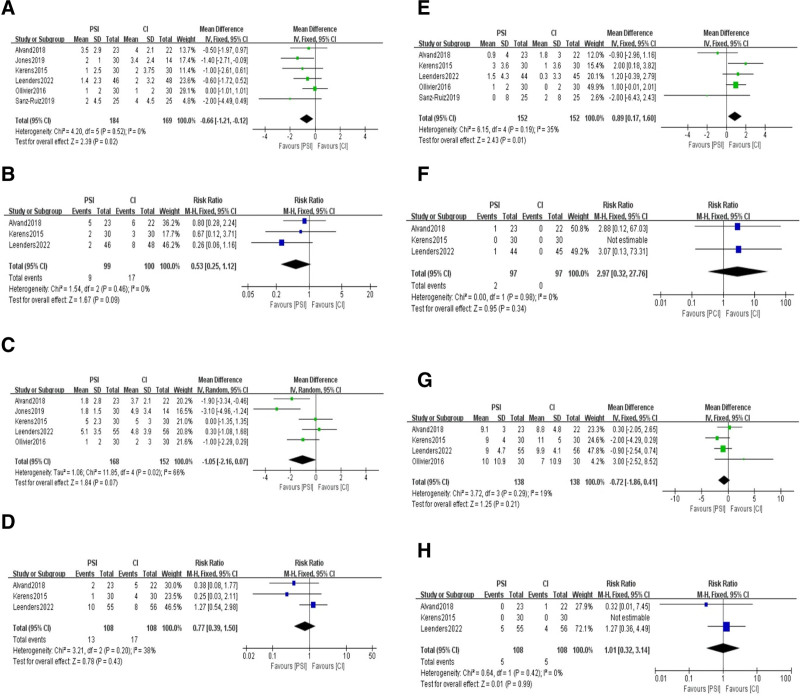
Postoperative tibial coronal alignment in the PSI and CI groups: (A) absolute deviation from the target alignment and (B) number of outliers (>3º from the target alignment). Postoperative tibial sagittal alignment in the PSI and CI groups: (C) absolute deviation from the target alignment and (D) number of outliers (>3º from the target alignment). Postoperative femoral coronal alignment in the PSI and CI groups: (E) absolute deviation from the target alignment and (F) number of outliers (>3º from the target alignment). Postoperative femoral sagittal alignment in the PSI and CI groups: (G) absolute deviation from the target alignment and (H) number of outliers (>3º from the target alignment). PSI = patient-specific instrumentation.

Six studies^[19,22,24–27]^ compared the postoperative tibial coronal alignment as the mean and standard deviation. The results of the heterogeneity test indicated low heterogeneity among the studies (*I*² = 0). A fixed-effects model was used for the meta-analysis, which showed a significant difference in tibial coronal alignment between the 2 surgical methods (MD = −0.66, 95% CI: −1.21 to −0.12, *P* = .02) (Fig. 3A). Three studies^[19,24,25]^ compared the outliers of postoperative tibial coronal alignment. The heterogeneity test results indicated low heterogeneity among the studies (*I*² = 0). The results of the meta-analysis indicate that there is no statistically significant difference in outliers of tibial coronal alignment between the 2 surgical approaches (RR = 0.53, 95% CI: 0.25–1.12, *P* = .09) (Fig. 3B).

### 3.4. Postoperative tibial sagittal alignment and number of outliers

Five studies^[19,22,24,25,27]^ compared the postoperative tibial sagittal alignment as the mean and standard deviation. The results of the heterogeneity test indicated high heterogeneity among the studies (*I*² = 66%). A random-effects model was used for the meta-analysis, which showed no statistically significant difference in tibial sagittal alignment between the 2 surgical methods (MD = −1.05, 95% CI: −2.16 to 0.07, *P* = .07) (Fig. 3C). Three studies^[19,24,25]^ compared the outliers of postoperative tibial sagittal alignment. The heterogeneity test results indicated low heterogeneity among the studies (*I*² = 38%). A fixed-effects model was used for the meta-analysis, which showed no statistically significant difference in outliers of tibial sagittal alignment between the 2 surgical approaches (RR = 0.77, 95% CI: 0.39–1.50, *P* = .43) (Fig. 3D).

### 3.5. Postoperative femoral coronal alignment and number of outliers

Five studies^[19,24–27]^ compared the postoperative femoral coronal alignment as the mean and standard deviation. The results of the heterogeneity test indicated low heterogeneity among the studies (*I*² = 35%). A fixed-effects model was used for the meta-analysis, which showed a significant difference in femoral coronal alignment between the 2 surgical methods (MD = 0.89, 95% CI: 0.17–1.60, *P* = .01) (Fig. 3E). Three studies^[19,24,25]^ compared the outliers of postoperative femoral coronal alignment. The heterogeneity test results indicated no heterogeneity among the studies (*I*² = 0%). A fixed-effects model was used for the meta-analysis, which showed no statistically significant difference in outliers of femoral coronal alignment between the 2 surgical approaches (RR = 2.97, 95% CI: 0.32–27.76, *P* = .34) (Fig. 3F).

### 3.6. Postoperative femoral sagittal alignment and number of outliers

Four studies^[19,24,25,27]^ compared the postoperative femoral sagittal alignment as the mean and standard deviation. The results of the heterogeneity test indicated low heterogeneity among the studies (*I*² = 19%). A fixed-effects model was used for the meta-analysis, which showed no statistically significant difference in femoral sagittal alignment between the 2 surgical methods (MD = −0.72, 95% CI: −1.86 to 0.41, *P* = .21) (Fig. 3G). Three studies^[19,24,25]^ compared the outliers of postoperative femoral sagittal alignment. The heterogeneity test results indicated no heterogeneity among the studies (*I*² = 0%). A fixed-effects model was used for the meta-analysis, which showed no statistically significant difference in outliers of femoral sagittal alignment between the 2 surgical approaches (RR = 1.01, 95% CI: 0.32–3.14, *P* = .99) (Fig. 3H).

### 3.7. Postoperative complications and surgery time

**Figure 4.**
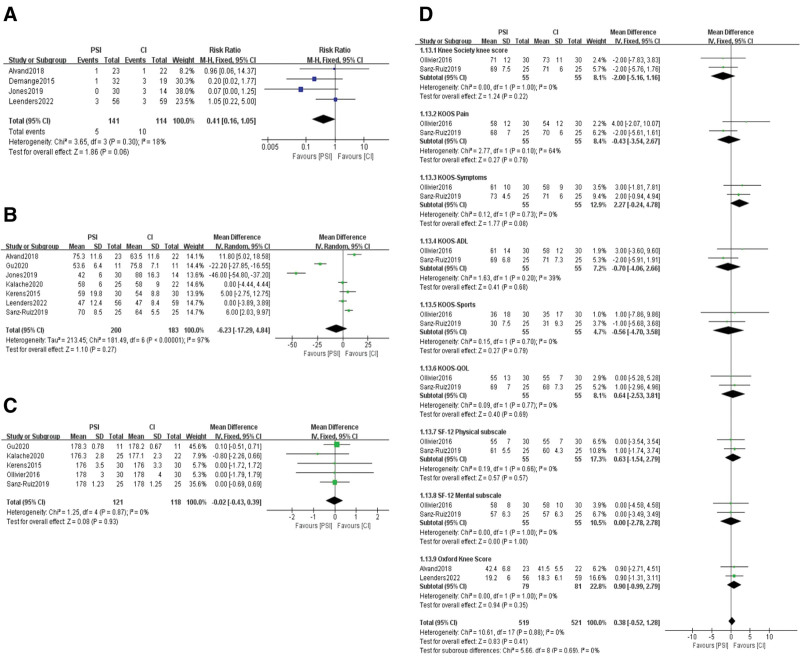
(A) Postoperative complications with PSI versus SI. (B) Operative time with PSI versus SI. (C) HKA angle with PSI versus SI. (D) Postoperative knee function with PSI versus SI. PSI = patient-specific instrumentation.

Four studies^[19,20,22,25]^ reported postoperative complications, with 5 cases in the PSI group and 10 cases in the CI group. A fixed-effects model was used for the meta-analysis and the results showed no statistically significant difference between the 2 groups (RR = 0.41, *95%* CI: 0.16–1.05, *P = *.06) (Fig. 4A). A total of 7 studies^[19,21–26]^ reported the surgical duration for the 2 groups, with 200 cases in the PSI group and 183 cases in the CI group. The results of the heterogeneity test indicated high heterogeneity among the studies (*I*² = 97%) and random-effects model was used. There was no statistically significant difference in surgical duration between the 2 groups (MD = −6.23, 95% CI: −17.29 to 4.84, *P* = .27) (Fig. 4B).

### 3.8. HKA angle and postoperative knee joint function

Five studies^[21,23,24,26,27]^ reported the HKA angle, with 121 samples in the PSI group and 118 samples in the CI group. The meta-analysis results showed no significant difference in the HKA angle between the PSI and CI groups (MD = −0.02, 95% CI: −0.43 to 0.39, *P* = .93) (Fig. 4C). A total of 4 studies^[19,25–27]^ reported the postoperative knee joint function assessed by the 12-item Short-Form, the Knee Society Score, the Knee Injury and Osteoarthritis Outcome Score, and the Oxford Knee Score. The meta-analysis results showed no statistically significant heterogeneity in the effect sizes of the included studies (*I*² = 0%). Using a fixed-effects model, there was no statistically significant difference between the 2 groups (MD = 0.38, 95% CI: −0.52 to 1.28, *P* = .69) (Fig. 4D).

## 4. Discussion

During conventional knee joint replacement with bone resection, different sizes of trial components are compared with the patient bone size to determine the appropriate prosthesis. Poor prosthesis positioning is considered a factor contributing to early failure in UKA.^[28,29]^ However, prosthesis positioning generally relies on the surgeon experience.^[30]^ PSI offers advantages such as preoperative precise measurements, intraoperative personalized bone resection, and accurate placement of the prosthesis, theoretically enabling more accurate alignment and postoperative outcomes.^[31–33]^ Although there have been some studies comparing PSI with CI in recent years, there is still debate regarding whether PSI can achieve better lower limb alignment and postoperative outcomes.^[5,34,35]^ Therefore, in light of this, we conducted a meta-analysis to further clarify the advantages and limitations of PSI compared to CI, aiming to assist clinical physicians in selecting appropriate approaches to facilitate rehabilitation.

It is well-established that lower limb alignment and implant positioning are crucial factors influencing surgical success.^[28,36]^ We compared the PSI and CI groups in terms of lower limb alignment and implant positioning, and found that the PSI confer an advantage in achieving superior tibial component coronal alignment, whereas the CI associated with better femoral component coronal alignment. Clearly this result suggests that PSI may not be as reliable as theorized in reducing disparities in lower limb alignment and implant positioning. These disappointing results can be attributed to several factors, one of which may be related to the assessment of post-operative alignment. The routine evaluation of post-operative alignment is typically done using X-ray imaging due to reasons such as radiation exposure, while CT scans are less common. Therefore, the assessment of rotational angles is limited.^[24,25]^ Another factor that may influence the results is the learning curve.^[26,37]^ The clinical application of PSI is still in its exploratory stage, and the familiarity of surgeons with different procedures can impact the aforementioned factors to some extent. The current findings are consistent with recent research, suggesting that PSI may not be as reliable as initially believed.

We further compared the differences in surgical time between the 2 groups and found no statistically significant disparities. Despite the elimination of volume and complexity associated with conventional instruments, PSI did not demonstrate a clear advantage in reducing surgical time. This could be attributed to the fact that PSI is a new technology, and the familiarity of the operating physician may play a role. Subsequently, we compared the differences between the 2 groups in terms of functionality and safety. The research results indicate that there were no significant differences in postoperative functionality and safety between patients who received PSI and those who received CI. It is worth noting that only 4 studies provided the aforementioned information, and further research is needed to validate these findings.

Currently, PSI is in the preliminary exploration stage, and clarifying its differences compared to traditional surgery would help us choose appropriate approaches to assist patients’ recovery. This study has yielded 3 key findings: Firstly, we compared the PSI and CI groups in terms of lower limb alignment and implant positioning, and found that the PSI confer an advantage in achieving superior tibial component coronal alignment, whereas the CI associated with better femoral component coronal alignment, indicating that PSI may not be as reliable as theorized in reducing disparities in lower limb alignment and implant positioning. Secondly, PSI did not demonstrate a time-saving advantage. Thirdly, compared to traditional methods, PSI showed no significant differences in safety and postoperative functionality. These findings indicate that PSI does not possess evident advantages over traditional methods. Therefore, in clinical practice, it is important to select the appropriate surgical approach based on patient conditions and cost factors. Additionally, these findings further suggest the need to develop new technologies, such as robot-assisted techniques, to reduce postoperative revision rates and improve postoperative functionality.

There are inevitably some limitations in this meta-analysis. Firstly, the included studies encompassed PSI systems from multiple manufacturers, which introduces potential bias. Due to limited data availability, subgroup analysis for different PSI systems could not be conducted. Secondly, the meta-analysis of functional outcome scores lacks sufficient data support. Thirdly, certain data transformations performed in the study may have impacted the analysis of results. Fourth, the duration of follow-up in the included studies was relatively short, and the long-term therapeutic efficacy outcomes remain uncertain. Furthermore, the substantial heterogeneity among the studies included in some of the results may affect the robustness of the findings. Considering the combined impact of these limitations, caution should be exercised when drawing conclusions from this study.

## 5. Conclusions

Our study findings suggest that the PSI confer an advantage in achieving superior tibial component coronal alignment, whereas the CI associated with better femoral component coronal alignment. However, our analysis did not reveal any significant differences between the groups regarding other parameters and outcomes. Further research is warranted to explore additional factors that might impact these outcomes and assess the long-term effects and potential risks associated with these surgical techniques.

## Author contributions

**Software:** Liang Zhang.

**Writing –original draft:** Huihui Wang.

**Writing –review & editing:** Xueren Teng.

## Supplementary Material


