# Supplementary material for: The efficacy and safety of patient-specific instrumentation versus conventional instrumentation for unicompartmental knee arthroplasty: Evidence from a meta-analysis
Source: Medicine (Baltimore). 2024 Jan 19;103(3):e36484. doi: 10.1097/MD.0000000000036484 (PMC10798727; doi:10.1097/MD.0000000000036484)
Supplement: Supplementary file 1 [file medi-103-e36484-s001.docx]

**Table S1: Electronic Search strategy**

| PubMed |  |
| --- | --- |
| Search Items | Results |
| ("printing, three dimensional"[MeSH Terms] OR ("printing"[All Fields] AND "three dimensional"[All Fields]) OR "three-dimensional printing"[All Fields] OR ("three"[All Fields] AND "dimensional"[All Fields] AND "printing"[All Fields]) OR "three dimensional printing"[All Fields] OR "3D-printed"[All Fields] OR (("patient s"[All Fields] OR "patients"[MeSH Terms] OR "patients"[All Fields] OR "patient"[All Fields] OR "patients s"[All Fields]) AND ("sensitivity and specificity"[MeSH Terms] OR ("sensitivity"[All Fields] AND "specificity"[All Fields]) OR "sensitivity and specificity"[All Fields] OR "specificity"[All Fields] OR "specific"[All Fields] OR "specifically"[All Fields] OR "specification"[All Fields] OR "specifications"[All Fields] OR "specificities"[All Fields] OR "specifics"[All Fields] OR "specifities"[All Fields] OR "specifity"[All Fields]) AND ("instrumentation"[MeSH Subheading] OR "instrumentation"[All Fields] OR "instrumentation s"[All Fields] OR "instrumentational"[All Fields] OR "instrumentations"[All Fields] OR "instrumention"[All Fields])) OR (("patient s"[All Fields] OR "patients"[MeSH Terms] OR "patients"[All Fields] OR "patient"[All Fields] OR "patients s"[All Fields]) AND ("match"[All Fields] OR "matched"[All Fields] OR "matches"[All Fields] OR "matching"[All Fields] OR "matchings"[All Fields])) OR (("customisable"[All Fields] OR "customisation"[All Fields] OR "customise"[All Fields] OR "customised"[All Fields] OR "customising"[All Fields]) AND ("instrumentation"[MeSH Subheading] OR "instrumentation"[All Fields] OR "instrumentation s"[All Fields] OR "instrumentational"[All Fields] OR "instrumentations"[All Fields] OR "instrumention"[All Fields])) OR (("culture"[MeSH Terms] OR "culture"[All Fields] OR "custom"[All Fields] OR "customs"[All Fields] OR "customer"[All Fields] OR "customer s"[All Fields] OR "customers"[All Fields] OR "customization"[All Fields] OR "customizations"[All Fields] OR "customize"[All Fields] OR "customized"[All Fields] OR "customizes"[All Fields] OR "customizing"[All Fields]) AND ("cutting"[All Fields] OR "cuttings"[All Fields]) AND ("block"[All Fields] OR "blocked"[All Fields] OR "blocking"[All Fields] OR "blockings"[All Fields] OR "blocks"[All Fields]))) AND ("arthroplasty, replacement, knee"[MeSH Terms] OR ("arthroplasty"[All Fields] AND "replacement"[All Fields] AND "knee"[All Fields]) OR "knee replacement arthroplasty"[All Fields] OR ("unicompartmental"[All Fields] AND "knee"[All Fields] AND "arthroplasty"[All Fields]) OR "unicompartmental knee arthroplasty"[All Fields] OR "UKA"[All Fields] OR ("arthroplasty, replacement, knee"[MeSH Terms] OR ("arthroplasty"[All Fields] AND "replacement"[All Fields] AND "knee"[All Fields]) OR "knee replacement arthroplasty"[All Fields] OR ("unicondylar"[All Fields] AND "knee"[All Fields] AND "replacement"[All Fields]) OR "unicondylar knee replacement"[All Fields])) AND ("clinical trial"[Publication Type] OR "clinical trials as topic"[MeSH Terms] OR "clinical trial"[All Fields] OR ("randomized controlled trial"[Publication Type] OR "randomized controlled trials as topic"[MeSH Terms] OR "randomized controlled trial"[All Fields] OR "randomised controlled trial"[All Fields])) | 211 |

| Web of science |  |
| --- | --- |
| Search Items | Results |
| (((((TS=(three—dimensional printing)) OR TS=(3D-printed)) OR TS=(patient specific instrumentation)) OR TS=(patient matched)) OR TS=(customised instrumentation)) OR TS=(custom cutting block) | 353 |

| Cochrane Library | |  |
| --- | --- | --- |
| History | Search Items | Results |
| #1 | (three—dimensional printing):ti,ab,kw OR (patient specific instrumentation):ti,ab,kw OR (3D-printed):ti,ab,kw OR (patient matched):ti,ab,kw OR (customised instrumentation):ti,ab,kw | 40693 |
| #2 | (unicompartmental knee arthroplasty):ti,ab,kw OR (unicondylar knee replacement):ti,ab,kw OR (UKA):ti,ab,kw | 257 |
| #3 | #1 and #2 | 22 |

| Excerpta Medica Database (EMBASE) | |  |
| --- | --- | --- |
| History | Search Items | Results |
| #1 | ‘three—dimensional printing’:ti,ab,kw OR ‘patient specific instrumentation’:ti,ab,kw OR ‘3D-printed’:ti,ab,kw OR ‘patient matched’:ti,ab,kw OR ‘customised instrumentation’:ti,ab,kw | 20013 |
| #2 | ‘unicompartmental knee arthroplasty’:ti,ab,kw OR ‘unicondylar knee replacement’:ti,ab,kw OR ‘UKA’:ti,ab,kw | 2812 |
| #3 | #1 and #2 | 35 |
